# Potential Inaccuracies in Chloride Measurements in Patients with Severe Metabolic Acidosis

**DOI:** 10.1155/2012/768316

**Published:** 2012-06-12

**Authors:** Tetsuya Makiishi, Naomasa Nishimura, Keiko Yoshioka, Shinya Yamamoto, Ryuichi Mitsuhashi, Sayako Maeda, Takashi Konishi, Kunihiko Hirose

**Affiliations:** ^1^Division of Nephrology, Department of Internal Medicine, Otsu Red Cross Hospital, 1-1-35 Nagara, Shiga Otsu 520-8511, Japan; ^2^Department of Clinical Laboratory, Otsu Red Cross Hospital, 1-1-35 Nagara, Shiga Otsu 520-8511, Japan; ^3^Department of Cardiology, Otsu Red Cross Hospital, 1-1-35 Nagara, Shiga Otsu 520-8511, Japan

## Abstract

*Background*. To address the cause(s) of the significant differences in chloride (Cl^−^) concentrations between point-of-care blood gas analyzers and central laboratory analyzers. *Methods*. Cl^−^ concentrations measured simultaneously by a blood gas analyzer (ABL800 FLEX) and a central laboratory analyzer (Hitachi7600) were collected in patients with severe acidemia (pH < 7.20) (*n* = 32) and were examined for correlations between differences in Cl^−^ and factors associated with the acid-base status. Cl^−^ concentrations were measured with both analyzers for samples with different concentrations of lactate, inorganic phosphate, or bicarbonate (HCO_3_^   −^). *Results*. The differences in Cl^−^ concentrations were correlated with HCO_3_^   −^ concentrations (*r* = 0.72, *P* < 0.0001) and anion gap (*r* = 0.69, *P* < 0.0001). Only the addition of HCO_3_^   −^ proportionately increased Cl^−^ levels measured by a Hitachi7600, but it did not affect those measured by an ABL800FLEX. *Conclusion*. Cl^−^ measurements with some analyzers may be influenced by HCO_3_^   −^ concentrations, which could result in the observed discrepancies.

## 1. Introduction

Measurement of the serum chloride (Cl^−^) concentration is essential to assess a patient's acid-base status, because the Cl^−^ concentration is used to calculate the anion gap (AG) in patients with metabolic acidosis and the Cl^−^ deficit in patients with metabolic alkalosis [1, 2]. The Cl^−^ concentration is usually determined by a central laboratory-automated biochemical analyzer (central laboratory analyzer), although point-of-care blood gas and electrolyte analyzers (blood gas analyzer) are now available in many clinical facilities. 

 Recent studies have shown that electrolyte values determined by a central laboratory analyzer and a blood gas analyzer may differ [3–7]. In such studies, the sodium (Na^+^) concentrations measured by a central laboratory analyzer tend to be higher, while chloride (Cl^−^) concentrations tend to be lower compared with those measured by a blood gas analyzer, leading to significant differences in the AG as well as chloride deficit between these methods [3]. If the differences are large, they might hamper the clinician's assessment of the patient's acid-base status.

 Although the precise mechanisms for this phenomenon are unclear, the dilutional effect of heparin [4, 8] and its direct binding to electrolytes [4, 9] are thought to be responsible for the lower Na^+^ and potassium (K^+^) values determined by a blood gas analyzer. However, there are still few explanations for the lower Cl^−^ values determined by central laboratory analyzers than those determined by blood gas analyzers. We recently encountered a case with end-stage renal disease (ESRD) with marked discrepancies in Cl^−^ concentrations between the two methods, which improved following several sessions of hemodialysis. This case prompted the hypothesis that Cl^−^ concentrations measured by the analyzers are influenced by acid-base status.

## 2. Methods

The institutional ethics committee waived the need for informed consent for this anonymous retrospective chart analysis. Data were collected from staff at Otsu Red Cross Hospital as part of standard patient care.

### 2.1. Patients and Samples

#### 2.1.1. Patients Admitted to an Intensive Care Unit (ICU)

We retrospectively collected data from patients admitted to our ICU between April 1, 2010 and June 30, 2010, for whom samples were simultaneously measured by a blood gas analyzer and a central laboratory analyzer. In total, 48 pairs of values were collected from 19 patients, and the mean electrolyte concentrations were compared between the two analyzers. The patients' samples were then divided into two groups; those from acidic patients (pH < 7.40) and those from alkalemic patients (pH > 7.40), and we compared the mean differences in electrolyte concentrations measured with both analyzers for sets of samples.

#### 2.1.2. Patients with Severe Acidemia

We retrospectively collected data measured with the blood gas analyzer that showed severe acidemia (pH ≤ 7.20) in patients admitted to our hospital between April 1, 2010 and July 7, 2010. Among 102 datasets, 32 pairs of samples from 29 patients were simultaneously measured with both analyzers. We determined correlations between the differences in Cl^−^ concentrations and pH, pCO_2_, bicarbonate (HCO_3_^−^), and gAG, which were defined as follows: gAG = Na^+^(blood gas) − Cl^−^(blood gas) − HCO_3_^−^.

#### 2.1.3. Patients with ESRD

We retrospectively collected data from patients who started maintenance dialysis therapy for ESRD at our hospital between April 1, 2009 and June 30, 2010. Among 45 cases, 37 pairs of samples from 37 patients taken at the start of dialysis were simultaneously measured by the blood gas analyzer and the central laboratory analyzer. We determined correlations between the differences in Cl^−^ concentrations with HCO_3_^−^ concentrations and gAG.

#### 2.1.4. Patients with Lactic Acidosis

Among 32 pairs of samples used in the study of patients with severe acidemia, nine had lactate levels >5 mmol/L, serum creatinine levels <2 mg/dL, and serum glucose levels of <300 mg/dL. These samples were defined as having lactic acidosis and were used to determine correlations between the differences in Cl^−^ concentrations with the HCO_3_^−^ and lactate concentrations.

### 2.2. Sample Collection and Measurements

All blood samples were collected from arteries, except for samples obtained from patients with ESRD, which were collected from shunt vessels or hemodialysis catheters. Whole-blood samples collected with blood-gas syringes containing lithium-heparin (PrezaPack II; Terumo Co., Tokyo, Japan) were analyzed in a point-of-care blood gas analyzer (ABL800 FLEX; Radiometer Medical A/S, Bronshoj, Denmark) to determine blood gases, Na^+^, K^+^, Cl^−^, and lactate concentrations. It also determines the HCO_3_^−^ concentration from pH and pCO_2_ using the Henderson-Hasselbalch equation.

 For each patient, a paired sample was simultaneously drawn by the vacuum technique with a serum separator tube. The serum samples were analyzed by a central laboratory analyzer (Hitachi7600 clinical analyzer; Hitachi High Technologies Co., Tokyo, Japan) to measure multiple biochemical variables, which included creatinine, blood urea nitrogen (BUN), glucose, and electrolytes. The analyzers were checked daily, regularly inspected, and tested for accreditation in accordance with Japanese laboratory standards.

### 2.3. Experimental Studies

To explore the possible explanations of our findings, we also conducted experimental studies. To test the effect of anions on the measured Cl^−^ concentrations, we prepared a control sample with 3.5% albumin-acetate Ringer's solution and 105 mEq/L Cl^−^, but without HCO_3_^−^. The Cl^−^ concentration was determined as the mean of three measurements done using the blood gas analyzer (ABL800FLEX). Then, we measured the Cl^−^ concentrations three times in samples containing different concentrations of lithium lactate, potassium dihydrogen phosphate (KH_2_PO_4_), or sodium bicarbonate (NaHCO_3_) using a blood gas analyzer (ABL800FLEX) and a central laboratory analyzer (Hitachi7600). Samples containing 50 and 100 mg/dL lactate were prepared by adding 5.33 and 10.66 mg lithium lactate to a tube containing 10 mL of the control sample, while samples containing 10 and 20 mg/dL PO_4_^3−^ were prepared by adding 1.43 and 2.86 mg KH_2_PO_4_ to 10 mL of the control sample. The required concentrations of HCO_3_^−^ were prepared by adding 8.4, 16.8, 25.2, 33.6, 42.0, 50.4, 55.8, 67.2, 75.6, and 84.0 mg NaHCO_3_ to tubes containing 10 mL of the control sample.

### 2.4. Statistical Analyses

Statistical analyses were performed using SPSS version 17.0 (SPSS Inc., Chicago, IL, USA). Student's paired *t*-test was used to assess differences in electrolyte concentrations between the two analyzers. Unpaired *t*-tests were used to assess differences between mean differences in electrolyte concentrations measured with two analyzers between the acidemia and alkalemia groups. Pearson's product-moment correlation coefficients (*r*) were calculated to determine the relationships between Cl^−^ concentrations and other variables (gAG, lactate level, pH, and pCO_2_). One-way ANOVA followed by Tukey's post hoc test was used to assess the effects of lactate, PO_4_^3−^, and HCO_3_^−^ concentrations on the Cl^−^ concentrations measured using the ABL800FLEX and Hitachi7600. Estimates are given with 95% confidence intervals (CIs). Values of *P* < 0.05 were considered statistically significant.

## 3. Results

### 3.1. Associations in Patients Admitted to the ICU

This analysis comprised 48 pairs of samples from 19 patients aged 70.6 ± 11.3 years (10 males, 23 pairs of samples; 9 females, 25 pairs of samples). The mean biochemical values were as follows: arterial pH, 7.43 ± 0.05 (range, 7.25–7.55); PaCO_2_, 36.7 ± 6.1 mmHg (21.2–52.0 mmHg); HCO_3_^−^, 23.8 ± 3.2 mmol/L (8.9–28.5 mmol/L); gAG, 6.5 ± 2.6 mEq/L (2.5–16.1 mEq/L); lactate, 1.3 ± 1.0 mmol/L (0.5–6.2 mmol/L). 

 The mean Na^+^ concentrations determined by the blood gas analyzer and central laboratory analyzer were 132.5 ± 6.3 mmol/L and 133.9 ± 6.4 mmol/L, respectively (*P* < 0.0001), corresponding to a mean difference of −1.4 mmol/L (95% CI: −1.9, −0.9 mmol/L). The mean K^+^ concentrations were 3.9 ± 0.6 mmol/L and 4.2 ± 0.5 mmol/L, respectively (*P* < 0.0001), with a mean difference of −0.3 mmol/L (95% CI: −0.4, −0.2 mmol/L). The mean Cl^−^ concentrations were 102.2 ± 5.8 mmol/L and 101.0 ± 5.6 mmol/L (*P* = 0.003), with a mean difference of 1.2 mmol/L (CI: 0.4, 2.0 mmol/L).

 When the 48 pairs of samples were divided into two groups according to pH, the mean difference in Cl^−^ concentration was significantly higher in samples from acidic patients (pH < 7.40) than in samples from alkalemic patients (pH > 7.40), whereas there were no statistically significant differences in Na^+^ or K^+^ concentrations between these groups (Table 1).

### 3.2. Associations in Patients with Severe Acidemia

To investigate the factor(s) associated with the differences in Cl^−^ concentrations between acidic and alkalemic groups, we determined correlations between the difference in Cl^−^ concentrations and several factors associated with the acid-base status (i.e., pH, pCO_2_, gAG, and HCO_3_^−^) in patients with severe acidemia. This analysis comprised 32 pairs of samples from 29 patients aged 70.0 ± 15.5 years (21 males, 24 pairs of samples; 8 females, 8 pairs of samples). The mean biochemical values were as follows: arterial pH, 7.01 ± 0.16 (range, 6.65–7.20); PaCO_2_, 80.0 ± 35.6 mmHg (25.8–183.0 mmHg); HCO_3_^−^, 19.3 ± 8.8 mmol/L (6.9–35.8 mmol/L); gAG, 12.3 ± 6.7 mmol/L (1.5–24.5 mmol/L); lactate, 8.7 ± 7.2 mmol/L (0.3–20.9 mmol/L).

**Figure 1:**
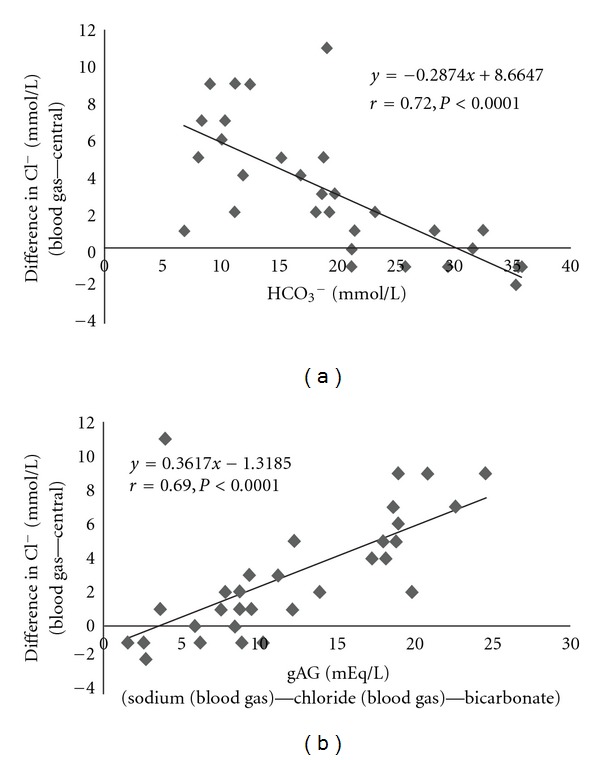
Correlations between the difference in chloride (Cl^−^) concentrations determined by two analyzers with bicarbonate (HCO_3_^−^) concentrations (a) and gAG (b) in patients with severe acidemia (pH < 7.2).

 In patients with severe acidemia, the difference in Cl^−^ concentration was well correlated with the HCO_3_^−^ concentration (*r* = 0.72, *P* < 0.0001) (Figure 1(a)). The difference also showed good correlations with gAG (*r* = 0.69, *P* < 0.0001) (Figure 1(b)), but weaker correlations with pH and pCO_2_ (*r* = 0.40, *P* = 0.02 and *r* = 0.43, *P* = 0.01, resp.).

### 3.3. Associations in Patients with ESRD and Lactic Acidosis

To further explore the possible relationship between the difference in Cl^−^ concentrations with HCO_3_^−^ concentrations and gAG, we determined correlations using data from several types of high-AG acidosis.

#### 3.3.1. ESRD

This analysis comprised 37 pairs of samples from 24 patients aged 70.3 ± 16.3 years (17 males, 28 pairs of samples; 7 females, 9 pairs of samples). The mean values were as follows: arterial pH, 7.31 ± 0.08 (range, 7.07–7.48); PaCO_2_, 34.7 ± 9.2 mmHg (13.7–54.9); HCO_3_^−^, 17.4 ± 5.7 mmol/L (7.9–32.2); gAG, 10.4 ± 4.0 mmol/L (2.8–23.8); lactate, 0.9 ± 0.3 mmol/L (0.8–1.7); BUN, 96 ± 35 mg/dL (38–212); serum creatinine, 7.88 ± 4.88 mg/dL (2.37–30.61). In data from 37 patients with ESRD, the differences in Cl^−^ concentrations were correlated with HCO_3_^−^ concentrations (*r* = 0.66, *P* < 0.0001) but not with gAGs (*r* = 0.24, *P* = 0.16).

#### 3.3.2. Lactic Acidosis

**Figure 2:**
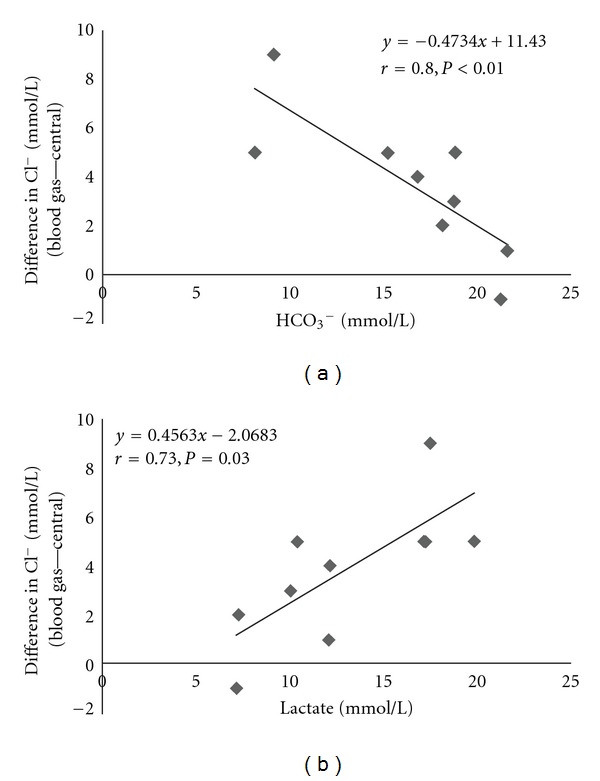
Correlations between the difference in chloride (Cl^−^) concentrations determined by two analyzers with bicarbonate (HCO_3_^−^) concentrations (a) and lactate concentrations (b) in patients with lactic acidosis.

The nine patients (8 males (89%) and 1 female (11%)) with lactic acidosis were 71.4 ± 17.5 years, with one pair of samples from each patient. The mean biochemical values were as follows: arterial pH, 6.99 ± 0.17 (range, 6.65–7.20); PaCO_2_, 74.9 ± 36.0 mmHg (25.8–146 mmHg); HCO_3_^−^, 16.4 ± 4.8 mmol/L (8.1–21.6 mmol/L); gAG, 14.0 ± 4.3 mmol/L (8.8–18.9 mmol/L); lactate, 12.6 ± 4.6 mmol/L (7.1–19.8 mmol/L); BUN, 25.2 ± 13.2 mg/dL (12.8–55.9 mg/dL); serum creatinine, 1.30 ± 0.47 mg/dL (0.38–1.89 mg/dL); serum glucose, 143 ± 62 mg/dL (47–227 mg/dL). In these patients, the differences in Cl^−^ concentrations were significantly correlated with HCO_3_^−^ (*r* = 0.80, *P* < 0.01) (Figure 2(a)) and lactate concentrations (*r* = 0.73, *P* = 0.03) (Figure 2(b)).

### 3.4. Experimental Studies

**Figure 3:**
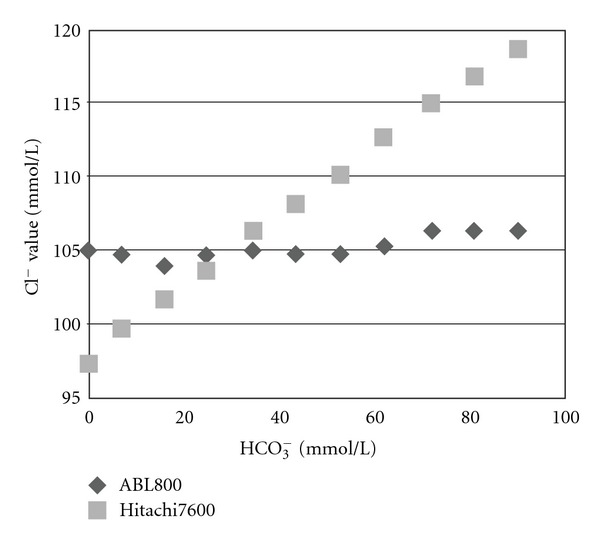
Interference of bicarbonate (HCO_3_^−^) concentrations on chloride (Cl^−^) concentrations measured with a Hitachi7600 or an ABL800. There was a concentration-dependent increase in Cl^−^ concentrations measured with the Hitachi7600 (i.e., the mean concentrations were statistically different from each other) but not with the ABL800, with increasing HCO_3_^−^ concentrations. Data are means.

In our experimental studies performed to understand the possible explanations for our findings, the addition of lithium lactate or KH_2_PO_4_ did not affect the Cl^−^ concentrations measured by both analyzers (Table 2). In contrast, the addition of NaHCO_3_ proportionately increased the Cl^−^ value measured by the Hitachi7600 but not the Cl^−^ value measured by the ABL800FLEX (Figure 3). The addition of NaHCO_3_ inevitably increased the pH and PCO_2_ values in each sample. The pH and PCO_2_ values increased proportionately from 7.226 ± 0.02 and 17.7 ± 0.2 mmHg, respectively, at an HCO_3_^−^ concentration of 7.1 ± 0.1 mmol/L to 7.964 ±0.01 and 36.0 ± 0.2 mmHg, respectively, at an HCO_3_^−^ concentration of 90.3 ± 0.3 mmol/L.

### 3.5. Magnitude of Difference between AGs Calculated from Values Measured by Two Analyzers

Clinically significant differences were found between AGs calculated with electrolyte values measured with ABL800FLEX and Hitachi7600 for samples from patients with severe acidemia, ESRD, and lactic acidosis (Table 3).

## 4. Discussion

The present study revealed that there are clinically significant differences in Cl^−^ concentrations measured using a central laboratory analyzer compared to those measured using a blood gas analyzer and that the differences were significantly correlated with HCO_3_^−^ concentrations and gAGs. The present study also revealed that HCO_3_^−^ concentrations are correlated with the Cl^−^ concentrations measured by the central laboratory analyzer but not with those measured using the blood gas analyzer. However, the addition of lactate or PO_4_^3−^ did not affect the Cl^−^ concentrations measured by either analyzer. These findings suggest that the Cl^−^ concentrations measured by some analyzers may significantly deviate from the real value in patients with severe acidosis or severe alkalosis, and these results may not be suitable for assessing a patient's acid-base status, such as the AG or the Cl^−^ deficit.

 Recent studies have reported small but significant differences in electrolyte concentrations measured using a point-of-care analyzer and a central laboratory analyzer [3–7]. In these studies, the mean Na^+^ [3–6] and K^+^ [5] concentrations were higher, while the mean Cl^−^ concentrations were lower when measured using a central laboratory analyzer compared with a blood gas analyzer [3, 6, 7]. Similar results were observed in the present study. There are several possible explanations for these differences in electrolyte concentrations. First, the sample preparations were different. Whole-blood samples were used for the blood gas analyzer whereas serum samples were used for the central laboratory analyzer. The sample tubes used for the blood gas analyzer contained solid-phase heparin, which could bind the electrolytes, thereby lowering the electrolyte concentrations measured by the blood gas analyzer [9]. The lower K^+^ concentrations determined using the blood gas analyzer in the present study may be explained by the use of an anticoagulant in the sample tubes used for the blood gas analyzer to prevent platelet rupture and the release of K^+^ into plasma [10]. Although these explanations could account for the lower Na^+^ and K^+^ concentrations, they do not account for the higher Cl^−^ concentrations measured with the blood gas analyzer.

 Interestingly, the mean differences in Cl^−^ concentrations between the two analyzers, but not Na^+^ and K^+^ concentrations, were significantly different between samples from acidic patients and those from alkalemic patients. Furthermore, the difference in Cl^−^ concentrations between the two analyzers was significantly correlated with HCO_3_^−^ concentration and gAG. These findings strongly suggest that the Cl^−^ concentrations determined with either of the two analyzers are influenced by factor(s) associated with the acid-base status.

 The Cl^−^ concentration is measured by an ion-selective electrode (ISE) method [11, 12]. Direct methods are usually used in blood gas analyzer, while indirect methods requiring predilution are used in central laboratory analyzer [6, 11]. Errors in the Cl^−^ concentration measured by the ISE method have been reported in several conditions. First, indirect methods may underestimate the Cl^−^ concentration because of dilution artifacts in patients with severe hypertriglyceridemia or dysproteinemia [11]. This is unlikely to explain our findings, because the mean Na^+^ concentrations measured by the central laboratory analyzer were higher than those measured by the blood gas analyzer. If dilution artifacts influenced the measurements, the Na^+^ concentrations measured by the central laboratory analyzer should be lower, not higher, than those measured by the blood gas analyzer. 

 Second, because the selectivity of the ISE method for Cl^−^ could be influenced by other anions present in the sample, it may interfere with the measurement in Cl^−^ concentration [11, 12]. The membranes of most currently used ISEs for Cl^−^ contain an ion-exchanger, a quaternary ammonium chloride. The selectivity of such membranes is in principle governed by the ion hydration energy. Hence, all ions that have hydration energy higher than or equivalent to Cl^−^ are considered as potentially interfering ions [13]. For example, cases of pseudohyperchloremia in patients with bromide (Br^−^) or iodide (I^−^) intoxication have been reported [14, 15]. It is unsurprising that both the HCO_3_^−^ concentrations and the gAGs showed good correlations with the difference in Cl^−^ concentrations in the present study because of an equimolar relationship between the HCO_3_^−^ concentration and the AG. These results suggest that HCO_3_^−^ or unmeasured anions such as lactate or PO_4_^−^, a major component of AG in patients with high AG acidosis, either alone or in combination, are responsible for the observed difference. In our experimental study, however, only the addition of HCO_3_^−^, a potential interfering ion [13], proportionately increased Cl^−^ levels measured by the central laboratory analyzer (Hitachi7600). This relationship indicates that HCO_3_^−^-mediated interference with the central laboratory analyzer may be responsible for the differences in Cl^−^ concentrations between the two analyzers. The addition of HCO_3_^−^ inevitably increased the pH and PCO_2_ values in each sample. Therefore, the possibilities that pH and/or PCO_2_ may explain the results of this clinical study cannot be excluded.

Because of the performance of the ISE and the composition of the calibration liquid as well as the calibrating system used in each analyzer, the degree of interference of potential interfering ions on Cl^−^ measurement may vary among analyzers [13, 16, 17]. Interestingly, two of the three central laboratory analyzers used in the studies that revealed statistically significant differences in Cl^−^ concentrations between the two methods are products from the same manufacturer of equipment used in our study [3, 6]. In fact, HCO_3_^−^ interference with the ISE from the manufacturer has already been reported [18, 19]. 

 Taken together, it seems reasonable to conclude that the measurement of Cl^−^ concentrations using the central laboratory analyzer is influenced by HCO_3_^−^ levels. This interference will likely result in underestimation of Cl^−^ concentrations in patients with metabolic acidosis and overestimation in patients with metabolic alkalosis, thereby overestimating and underestimating the AG and Cl^−^ deficit, respectively. Patients in whom calculation of the AG or Cl^−^ deficit is needed are often critically ill, and precise measurement is required. For patients with severe acidosis or alkalosis, clinically significant deviation from the real value could occur, which may result in wrong treatment.

 Our study has several limitations. First, we measured electrolytes with only two analyzers. Therefore, the results may not apply to comparisons of other blood gas analyzers and central laboratory analyzers. However, the two analyzers under scrutiny are used by hundreds of laboratories in Western countries, meaning our observations are very relevant to medical practice in many institutions. Second, our findings could reflect imprecision of one or both analyzers. However, this is unlikely because we measured approximately 130 samples over 12 months, and the analyzers were checked daily and regularly inspected and tested for accreditation in accordance with Japanese laboratory standards. Both analyzers performed in accordance with the standards for the measurement of each analyte under study. Third, the number of samples used in the four groups (ICU, severe acidemia, ESRD, and lactic acidosis) may not provide sufficient enough power to detect other contributing factors to the deviations in Cl^−^ measurements other than HCO_3_^−^. Similarly, the experimental studies could not exclude the possibilities that pH and/or PCO_2_ may be explain the deviations observed. Finally, because we selected specific groups of patients, the results and interpretations are subject to selection bias. Nevertheless, our experimental study supports the notion that the acid-base status of a patient affects the degree of inaccuracy in Cl^−^ measurements for some analyzers.

## 5. Conclusions

In conclusion, our study revealed that the measurement of Cl^−^ concentrations by some analyzers may be influenced by HCO_3_^−^ concentrations, which may account for the reported discrepancy between values measured by point-of-care and central laboratory analyzers. The deviation in Cl^−^ concentration from the real value, particularly in patients with severe acidosis or alkalosis, could be clinically significant and may lead to incorrect interpretation of a patients' acid-base status. Clinicians, especially nephrologists who are specialists in the field of blood gas analysis, should be aware of potential deviations in Cl^−^ concentrations measured by some analyzers. This is particularly important when Cl^−^ concentrations are used to estimate a patient's acid-base status. Clinical and laboratory staff should be aware of this issue until they verify that this is not the case for the analyzers used at their institutions. 

## Figures and Tables

**Table 1:** Mean values and mean differences in electrolyte concentrations measured simultaneously by two analyzers for samples from acidic patients (pH < 7.40) or alkalemic patients (pH > 7.40).

	pH < 7.40	pH > 7.40	*P*
	(*n* = 11)	(*n* = 37)	
Na^+^ (mmol/L)			
Blood gas analyzer	135.7 ± 4.0	131.5 ± 6.5	
Central laboratory analyzer	137.0 ± 3.6	133.0 ± 6.7	
Mean difference	−1.27 ± 1.79	−1.46 ± 1.59	0.74

K^+^ (mmol/L)			
Blood gas analyzer	3.6 ± 0.5	4.0 ± 0.5	
Central laboratory analyzer	3.9 ± 0.6	4.3 ± 0.5	
Mean difference	−0.25 ± 0.17	−0.27 ± 0.20	0.68

Cl^−^ (mmol/L)			
Blood gas analyzer	105.5 ± 3.6	101.2 ± 6.0	
Central laboratory analyzer	102.5 ± 3.4	101.0 ± 6.0	
Mean difference	3.10 ± 4.23	0.62 ± 1.55	0.004

Values are means ± SD. Na^+^: sodium; K^+^: potassium; Cl^−^: chloride.

**Table 2:** Chloride concentrations measured by two analyzers in samples containing 3.5% albumin-acetate Ringer's solution supplemented with different ionic concentrations of lactate or phosphate.

Cl^−^ (mmol/L)	Lactate concentration (mg/dL)		
	0	50	100
ABL800FLEX^∗^	104.7 ± 0.6	103.7 ± 0.6	104.7 ± 1.2
Hitachi7600^∗^	99.0 ± 0.1	97.5 ± 0.2	98.5 ± 0.6

	PO_4_^3−^ concentration (mg/dL)		
	0	10	20

ABL800FLEX^∗^	104.7 ± 0.6	105.3 ± 0.6	104.3 ± 0.6
Hitachi7600^∗^	99.0 ± 0.1	98.1 ± 0.3	97.2 ± 0.3

Values are means ± SD. *P > 0.05 for comparisons across lactate/PO_4_^3−^ concentrations. Cl^−^: chloride; PO_4_^3−^: phosphate.

**Table 3:** Differences in anion gap (AG) between the two analyzers for samples from patients with severe acidemia, end-stage renal disease, and lactic acidosis.

Patient cohort	Difference in AG (cAG − gAG)

Severe acidemia (*n* = 32)	5.0 ± 4.0 (1.0–24.0)

End-stage renal disease (*n* = 37)	4.6 ± 3.8 (1.0–24.0)

Lactic acidosis (*n* = 9)	6.3 ± 3.9 (1.0–13.0)

Values are means ± SD (range). cAG = Na^+^ (Central analyzer) − Cl^−^ (Central analyzer) − bicarbonate (HCO_3_^−^), gAG = Na^+^ (Blood gas analyzer) − Cl^−^ (Blood gas analyzer) −  HCO_3_^−^.
